# First Study of Mercury Content in Archaeological Pottery: Late-Neolithic Penha-Type from NW Spain

**DOI:** 10.3390/molecules31132335

**Published:** 2026-07-03

**Authors:** Antonio Martínez Cortizas, Ainé Francos Golán, Pilar Prieto Martínez, Olalla López-Costas

**Affiliations:** 1 EcoPast (GI-1553), Facultade de Bioloxía, Universidade de Santiago de Compostela, E-15782 Santiago de Compostela, Spain; 2 Bolin Centre for Climate Research, Stockholm University, 10691 Stockholm, Sweden; 3 EcoPast (GI-1553), Cross-Disciplinary Research Center on Environmental Technologies (CRETUS), Área de Arqueoloxía, Departamento de Historia, Universidade de Santiago de Compostela, 15782 Santiago de Compostela, Spain; aine.francos.golan@usc.es; 4 Archaeological Research Laboratory, Stockholm University, Wallenberglaboratoriet, 10691 Stockholm, Sweden; 5 EcoPast (GI-1553), Interuniversity Research Center for Atlantic Cultural Landscapes (CISPAC), Facultade de Xeografía e Historia, Universidade de Santiago de Compostela, 15782 Santiago de Compostela, Spain; pilar.prieto@usc.es

**Keywords:** ceramic, Penha-type, Hg, Neolithic, organic matter, kaolinite, iron, FTIR-ATR

## Abstract

In soils, mercury is found bound to organic matter, clays, and iron/manganese oxides, which are also major constituents of archaeological pottery. Although pottery is the most researched cultural material with archaeometric techniques, its mercury content remains largely unexplored. To address this gap, we studied Late Neolithic Penha-type pottery from NW Spain. The Late Neolithic was a period of widespread exploitation and circulation of mercury-bearing resources. A total of 92 samples from five archaeological sites were analysed to determine their mercury, carbon, sulfur, and iron content, as well as their spectroscopic properties (FTIR-ATR). Mercury was detected in all samples, with concentrations ranging from 6 to 1086 ng g^−1^. Neither organic matter (C and S) nor iron compounds (Fe) were found to explain Hg concentrations, suggesting that diagenetic mercury incorporation was unlikely. Mercury was found to be related to kaolinite structural transformations, with concentrations decreasing with increasing degree of transformation. Kaolinite transformation depended on firing conditions (temperature and time), pointing to thermal desorption of the mercury present in the clay. The large observed variability most probably resulted from poorly controlled firing conditions. Nevertheless, whether mercury content reflects unintentional incorporation from naturally mercury-rich raw materials or a deliberate selection or addition (e.g., of cinnabar) during pottery manufacture remains to be further explored.

## 1. Introduction

Pottery is the most abundant and accessible artefact in Archaeology [1], together with lithics. Investigations on its production from the Neolithic onwards, use, re-use or disposal are at the core of Archaeology and provide crucial contributions to the knowledge of ancient societies [2]. Its widespread availability and significant value have led to a considerable development of ceramic archaeometric research (among many others [2,3,4,5,6]), being ceramics a pioneering material [7], long before the arrival of processual Archaeology (amongst others [8]). Interests evolved from understanding how vessels were made, i.e., Chaîne Opératoire [9,10], how they were used [11], the acquisition and transmission of technological knowledge [12], and the biography of the pot [13]. Most of the archaeometric literature is focused on addressing the chemical–mineralogical composition of the clays, temper, or paints/glazers, as a first step prior to developing interpretations regarding their classification, provenance, production technologies, trade routes, or economic exchange [14,15,16].

There is a wide variety of decisions that potters had to make when producing a vessel: the choice of raw materials, tools, energy, techniques and their sequence [17], and archaeometry is used to gain insights into those five aspects. Minerals usually researched include clay minerals such as kaolinite, illite, or montmorillonite, and coarse temper minerals such as quartz, feldspar, calcite or other carbonates; sometimes minor accessory minerals were included [18,19], along with strong firing temperature dependence [20]. Chemically, most ceramics are composed of eight major elements, O, Na, Mg, Al, Si, K, Ca, and Fe [18,21]. Their large abundance on Earth makes them less useful to identify pots’ processing and provenance. Trace elements, such as metals or rare earth elements—mainly found in the clay fraction—are here more decisive (as an example for Prehistoric Iberia [22,23,24,25,26,27]). Their presence is influenced by both the mineralogical structure of the clay and the weathering/sorption processes inherited from pre and post-depositional history [18]. Geographical origin of raw materials is addressed by pursuing trace metals such as Pb, Co, or As, e.g., [28,29], Sr and Pb isotopic composition, e.g., [30,31], and more frequently a combination of elemental, mineralogical, and petrographic composition, e.g., [32,33,34]. However, to our knowledge, Hg has not been analysed before in archaeological pottery.

Mercury is a potentially toxic element, rare on the Earth’s surface, and highly appreciated by past societies [35]. It can be found in liquid form or more commonly associated with sulfur in the form of cinnabar—a bright red mineral. Although mercury mines are scarce, several are present in the Iberian Peninsula [36], including Almadén in Central Spain—a mine district with the largest mercury concentration in the world, exploited since Prehistory (5300 cal BC) [37,38]. Mercury exhibits substantial adsorption to and coprecipitation with iron oxides [39,40,41,42]. Nevertheless, this metal is found commonly bonded to organic matter, particularly to reduced sulfur groups [43,44,45,46,47] and N-containing compounds [48], or even to carbonyl and phenol functional groups [49,50]. Mercury concentrations are usually in the order of tens to a few hundred nanograms per gram, unsuitable for most routine archaeometric techniques, such as XRF or ICP-MS. For this reason, research of mercury in artifacts and ecofacts remains relatively limited, even though there have been notable advances in mercury content in archaeological human remains [51,52]. In contrast, mercury research in natural archives has a long tradition; see a review in [53].

Elemental mercury is in gaseous form at room temperature, and most of its forms, whether organic or inorganic, can be desorbed by thermal treatment at relatively low temperatures, to the point that thermal desorption is used as a technique to study mercury speciation [54]. This property implies that mercury should, in principle, be absent or in very low concentration in thermally altered materials. As Anna O. Shepard [2] pointed out, mercury and cinnabar should disappear upon heating, and the vivid red colour of cinnabar vanished. However, certain chemical bonds can prevent mercury volatilization, as we recently demonstrated in cremated human remains [55].

Late Neolithic (c. 4000–3000 cal BC) and Chalcolithic (c. 3100–1900 cal BC) societies from the Iberian Peninsula (for chronology attribution see, e.g., [56]) displayed a high social connection to mercury and more specifically to cinnabar. Several sites, mainly from southern and eastern Iberia, show mortuary practices that include spillage of cinnabar over the deceased bodies [57,58,59,60]. Its use can be documented until the Bronze Age, for example in the Argar culture (c. 1700 cal BC), where it has been found mainly associated with female skeletons [61]. It has even been found in areas far from Iberia, such as the cinnabar residues found in vessels from Serbia [62] or its extensive use in the Maya culture in Mesoamerica [63]. Most of the contexts where cinnabar was found are megalithic burials, but it was also found in non-megalithic collective areas, artificial caves, and wall house decorations [64]. The ecstasy of cinnabar use extended until the Bronze Age (c. 2200–800 BC cal), many times associated with Bell Beaker contexts (c. 2600–1700 BC cal) [65].

The Late Neolithic in northwestern Spain (Galicia, c. 3100–2400 cal BC) (for chronology, see [66]) witnessed profound transformations regarding previous chronologies [67], including a reduction in the monumentality of funerary structures [68]. The sparse material culture and lack of analyses limit our understanding of the extent of mercury tradition in this region. Only the Neolithic megalith of Chan da Armada (Pontevedra) was analysed for the presence of cinnabar, obtaining positive results [69]. In contrast, the Late Neolithic is characterised by a specific type of pottery, the Penha type, a homogeneous-looking bowl-shaped pottery characterised by matte reddish, brownish and blackish pastes [67]. Three sub-typologies have been described: Decorated Penha, Plain Penha (i.e., non-decorated) and Bell Beaker imitation [67,70,71]. In our previous study on this type of pottery, no compositional differences were found between these subtypes. Most vessels were made with local raw materials, whether mafic or felsic, being fired at low temperature (600–900 °C) and for a relatively short time (<5 h) [72]. Given the peculiar characteristics of Penha pottery, the paucity of information concerning the Late Neolithic in northwestern Iberia, and the prominent role of mercury/cinnabar in Iberian coetaneous societies, the well-studied assemblage of Penha-type ceramics from NW Iberia is a well-suited case study to explore the role of mercury as a constituent element of pottery.

Our general aim is to address whether the examined pottery contains mercury and the factors (technological, social, natural) behind the Hg content. In particular, the aims of this study are as follows: (1) determine mercury concentrations in the pottery and its association to organic matter (C and S contents) and iron oxides (Fe content); (2) examine its connection with the pottery’s colour, redness in particular—potentially related to temperature; and (3) to evaluate its connection with heat-induced mineral transformations (using FTIR-ATR). In this work, we seek the unexpected by analysing mercury concentrations in archaeological ceramics. Our hypothesis is that mercury retention in Late Neolithic pottery would be controlled by thermal desorption depending on firing conditions (temperature and time). The present research aligns with our conceptual framework, integrating the Chaîne Opératoire and object biography to research prehistoric societies through archaeological, anthropological, and archaeometric perspectives.

## 2. Results

### 2.1. Organic Matter (C and S), Iron (Fe) and Mercury (Hg) Content

**Figure 1:**
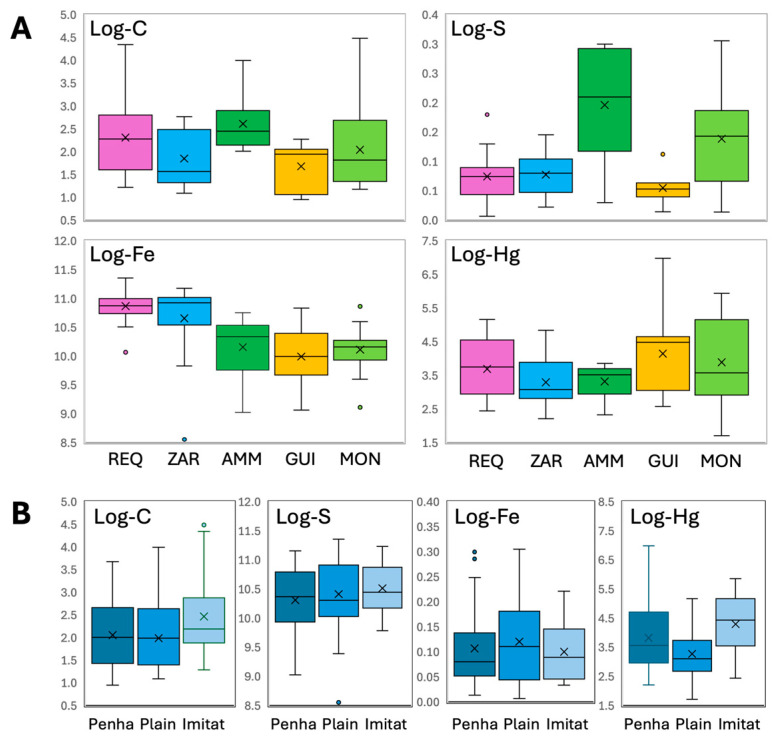
Box and whiskers plots of the log-transformed concentrations of C, S, Fe and Hg (for non-transformed values see Table A1 and Supplementary Materials). (**A**) Values by archaeological site (REQ, Requeán; ZAR, Zarra de Xoacín; AMM, As Mamelas; GUI, Guidoiro Areoso; MON, Montenegro). (**B**) Values grouped by pottery style (Penha, Penha-type decoration; Plain Penha, no decoration; Imitat, Bell Beaker decoration imitation).

Carbon, sulfur and iron content by archaeological site and pottery type can be found in Figure 1 (log-transformed data) and in Table A1 (raw data can also be found in Supplementary Materials). Average total carbon content varied between 2.61% and 1.68% (maximum 4.35% and minimum 0.95%). Differences between archaeological sites were significant (*p* = 0.020), following a sequence: ZAR, GUI ≤ REQ, MON ≤ AMM. Differences between pottery types were not significant (*p* = 0.136). Average sulfur (S) contents were low (0.20% to 0.05%), but differences between sites were also highly significant (*p* < 0.001), following essentially the same sequence as C content. Differences between pottery types were not significant (*p* = 0.29). Average iron (Fe) contents varied between 5.43% and 2.46% and showed highly significant differences (*p* < 0.001) between archaeological sites. The highest contents were found in samples from REQ and ZAR, sites which are located in areas of mafic rocks, and the lowest in samples from AMM, GUI, and MON, located in areas of felsic rocks. Differences between pottery styles were not significant (*p* = 0.443). Carbon, S, and Fe showed no significant correlations between them (Table 1).

Mercury concentrations varied within three orders of magnitude, ranging from 6 ng g^−1^ to 1086 ng g^−1^ (Table A1). Variation (i.e., difference between the minimum and the maximum values) was relatively low in AMM, REQ and ZAR, moderate in MON, and large in GUI. The highest average Hg concentration was recorded in the GUI samples (174 ± 344 ng g^−1^), and the lowest in AMM (30 ± 12 ng g^−1^) and ZAR (36 ± 33 ng g^−1^) (Table A1), but the differences between sites were not significant (*p* = 0.286). Despite this, differences between pottery styles were highly significant (*p* = 0.005): pots of Plain Penha showed the lowest Hg concentrations (40 ± 45 ng g^−1^), pots with Bell Beaker imitation decoration showed the highest values (107 ± 94 ng g^−1^), and pots with Penha decoration showed intermediate values (98 ± 174 ng g^−1^) (Figure 1, Table A1). Mercury concentration was not significantly correlated to C, S, or Fe concentrations (Table 1).

### 2.2. Colour (CIELab Space)

**Figure 2:**
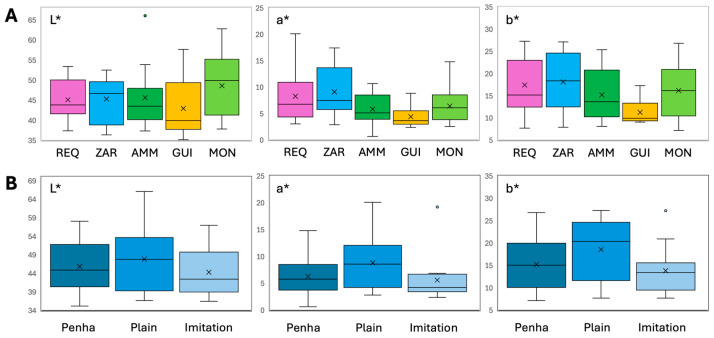
Box and whiskers plots of the colour parameters: L*, lightness; a*, redness; b*, yellowness. (**A**), values by archaeological site (REQ, Requeán; ZAR, Zarra de Xoacín; AMM, As Mamelas; GUI, Guidoiro Areoso; MON, Montenegro). (**B**), values grouped by pottery style (Penha, Penha-type decoration; Plain Penha, no decoration; Imitation, Bell Beaker decoration imitation).

All analysed pottery samples showed low lightness (L*) values, most of them below 50, and differences between archaeological sites were not significant (*p* = 0.341), nor for pottery styles (*p* = 0.234). As for the colour components, a* and b* both showed positive values, indicating the colour of the samples is a combination of red (positive a* values) and yellow (positive b* values) (Figure 2A). Both a* and b* presented highly significant differences between archaeological sites (*p* < 0.001 and *p* = 0.004)) and pottery styles (*p* = 0.008 and *p* = 0.014); sites located in areas of mafic rocks (REQ and ZAR) had higher values than those located in areas of felsic rocks (AMM, GUI, MON) (Figure 2A, Table A1). Regarding the style (i.e., subtypologies), Plain Penha pottery showed larger values than those of the other two styles (Figure 2B).

**Figure 3:**
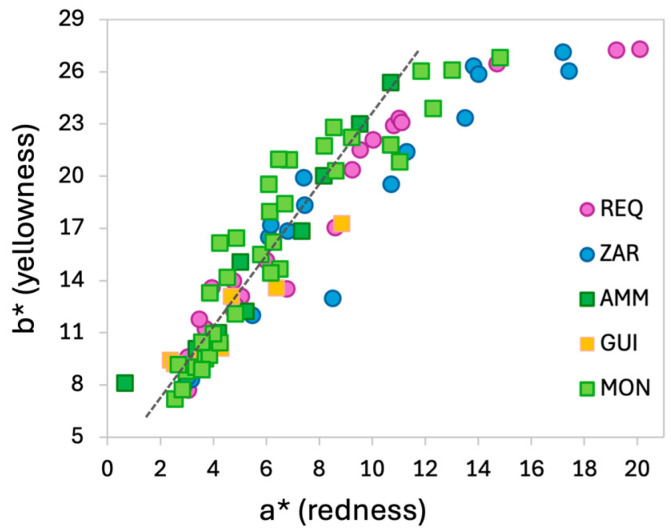
Projection of the values of CIELab colour components (a* and b*) for the Penha-type pottery samples of this study. The dashed line indicates the main trend of increasing chromatic intensity.

Almost all samples are distributed in a single trend of increasing a* and b* values, thus essentially representing changes in chromatic intensity but the same basic colour (Figure 3). Parameters a* and b* were highly correlated between them, while L* was highly correlated to b* but moderately correlated to a* (Table 1). For a* values greater than 10, the yellow component did not change, indicating a reddening not accompanied by changes in yellowness. Almost all samples departing from the trend (9 out of 12) are Plain Penha. Regarding the relation to the elemental composition, L* and a* were found to be moderately negatively correlated to C content. Correlation between a* and C, although significant, was marginal (Table 1). Correlation between a* (redness) and b* (yellowness) with Fe content was also marginal. No significant correlation to Hg was found.

### 2.3. Clay Transformations (Kaolinite and Talc)

**Figure 4:**
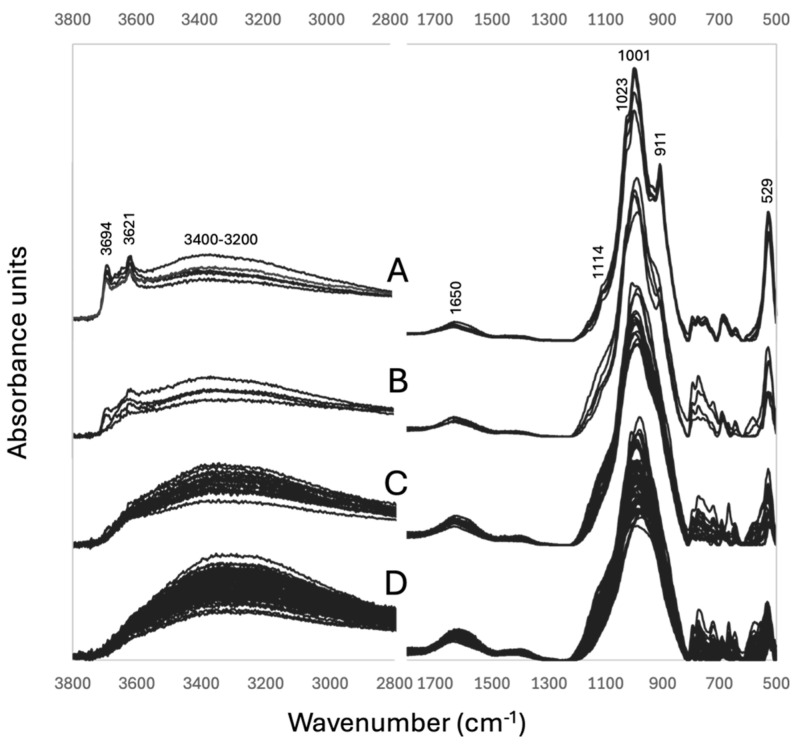
FTIR-ATR spectra of the studied Penha-type pottery samples from NW Spain. Spectra have been grouped based on the expression of the kaolinite peaks. A, well expressed (i.e., well preserved kaolinite); B to D, progressive collapse of the hydroxyl bands (3694, 3621, 911 cm^−1^) and reduction in the Si-O (1023, 1001 cm^−1^) and Al-O-Si (529 cm^−1^) vibration intensities.

The spectra of the studied samples are represented in Figure 4. A few samples (four from MON; Figure 4A) showed well-expressed peaks at 3694, 3621, 1114, 1023, 1001, 911 and 529 cm^−1^, related to OH stretching and deformation of surface and inner OH, Si-O stretching, and Al-O-Si deformation vibrations of kaolinite [73]. A second group of samples (one from AMM, one from GUI, one from MON, and one from REQ) still showed surface and inner OH stretching vibrations, but moderately expressed, and a decreased in intensity of the main Si-O (1023 and 1001 cm^−1^) band, the OH deformation band (911 cm^−1^), and the Al-O-Si band (529 cm^−1^) (Figure 4B). At the same time, the Si-O stretching (longitudinal mode) at 1114 cm^−1^ transformed into a shoulder that extends to 1200 cm^−1^ (Figure 2B). A third group of samples (two from AMM, 10 from MON, five from REQ and one from ZAR) only preserved a small shoulder assigned to the inner OH stretching vibration (3621 cm^−1^), while the OH deformation vibration (911 cm^−1^) almost disappeared and the Al-O-Si vibration further decreased (Figure 4C). In the spectra of the largest group of samples (seven from AMM, eight from GUI, 19 from MON, 15 from REQ, and 16 from ZAR), kaolinite OH vibrations completely disappeared, and the main Si-O absorbances and Al-O-Si absorbance further attenuated (Figure 4D).

Apart from the vibrations already described, moderate to weak absorbances were also detected in the 800–700 cm^−1^ region, which have been attributed to primary silicates (mainly quartz, K-feldspar, amphibole, mica, and plagioclase) as well as to secondary minerals (i.e., talc) in a recent investigation on these pottery samples [72].

**Figure 5:**
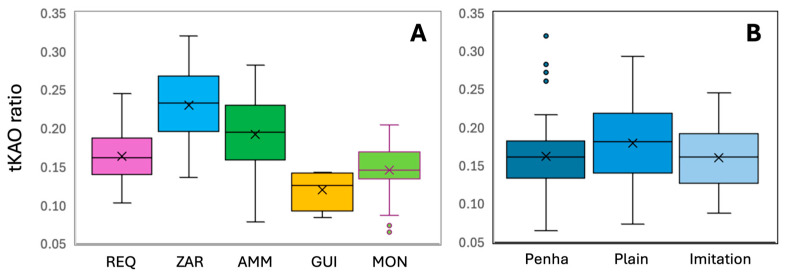
Box and whiskers plots of tKAO ratio (see material and methods). (**A**), values by archaeological site (REQ, Requeán; ZAR, Zarra de Xoacín; AMM, As Mamelas; GUI, Guidoiro Areoso; MON, Montenegro). (**B**), values grouped by pottery style (Penha, Penha-type decoration; Plain, Penha no decoration; Imitation, Bell Beaker decoration imitation).

In parallel to the changes observed in kaolinite absorbances, vibrations related to adsorbed water (OH stretching at 3400–3200 cm^−1^, and OH deformation at 1650 cm^−1^) progressively increased. We calculated a MIR index accounting for the degree of structural transformation of the kaolinite (tKAO; see Section 4). This index was defined as the ratio between the maximum absorbance intensity in the 3400–3200 cm^−1^ region and the maximum peak intensity of the main Si-O absorbances (1002 cm^−1^). The minimum tKAO value was 0.07 and the maximum 0.32. Differences between sites were highly significant (*p* < 0.001): samples from sites located in areas of felsic rocks (GUI, AMM, and MON) showed lower tKAO values than those of sites located in areas of mafic rocks (REQ and ZAR), except for AMM (Figure 5A). No significant differences (*p* = 0.332) were found for pottery style, i.e., Penha decoration, Plain and Bell Beaker imitation (Figure 5B).

**Figure 6:**
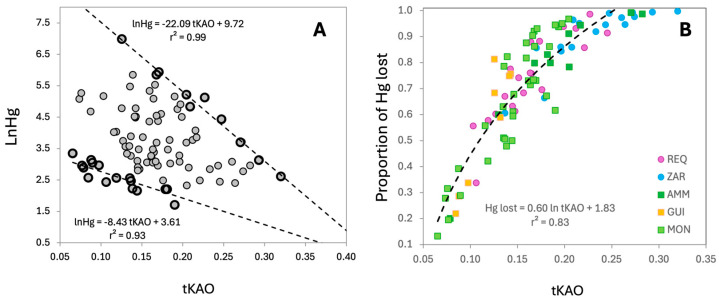
(**A**), Correlation between Hg (log-transformed) concentrations and the degree of structural transformation of kaolinite (tKAO). Samples enhanced with black circles were used to fit the upper and lower limits of the decrease in Hg concentration. (**B**), correlation between the degree of kaolinite structural collapse and the proportion of mercury lost during firing. Dash lines correspond to the fitting of the regression functions (linear in (**A**) and logarithmic in (**B**)).

The overall correlation between (log-transformed) Hg concentrations and tKAO was also low and not significant (r^2^ = 0.02), but values and variability in Hg concentration tend to decrease as tKAO increases, and samples are distributed between an upper and lower limit. Values of samples in the upper and lower limits can be fitted to linear regression lines, with very high determination coefficients (r^2^ > 0.90) (Figure 6A). Based on these results, the expected initial Hg concentration for samples in the upper limit would be 5517 ng g^−1^, and for samples in the lower limit, 37 ng g^−1^. Considering the actual Hg concentrations of the samples, this implies Hg losses of 13–99%. Assuming that the other samples also fit in individual trends, their expected initial Hg concentration was also calculated, and from that the proportion of Hg lost (Figure 6B). Mercury losses followed a logarithmic trend regarding the tKAO ratio (Figure 6B). Samples from ZAR and AMM sites had estimated losses greater than 80% (proportion > 0.8), while samples from MON and REQ covered the whole, or almost whole, range (13–99%), and samples from GUI showed values lower than 80%.

## 3. Discussion

### 3.1. Elemental Composition and Mercury Content

Significant differences in elemental composition (C, S, and Fe) were found between archaeological sites: GUI showed low contents for the three elements, whereas REQ and ZAR showed low contents for C and S, but high for Fe; AMM showed low Fe content but high C and S content, and MON showed low C and Fe contents, but relatively high S content.

Mercury concentrations also showed high variability, spanning up to two orders of magnitude both within and between sites. Although no Hg data are available for the clay fraction of soils from the study areas, data for the fine earth fraction (<2 mm) of topsoils are available elsewhere [74]. In soils from NW Spain developed on weathering products of felsic rocks, mercury concentrations range from 75 to 100 ng g^−1^, while in those developed on mafic materials concentrations are in the range 100–150 ng g^−1^, and clay contents vary between 10 and 20%. If we assume that Hg in these soils is bound to the finer colloidal fractions (i.e., clays), maximum concentrations in the clay fractions would be between 750 and 1500 ng g^−1^. These values are consistent with the maximum observed for the Penha pottery samples of our study.

Mercury is known to have a high affinity to bind to organic matter, preferentially to reduced sulfur groups [43,44,45,46,47] and N-containing compounds [48] and, to a lower extent, to carbonyl and phenol functional groups [49,50]. Mercury is also intensively adsorbed/coprecipitated with iron oxides [39,40,41,42]. Despite this, mercury content in the analysed pottery samples showed no significant correlation to total C and S (i.e., organic matter), nor to Fe (i.e., iron-bearing minerals). If Hg was to be adsorbed by the pottery through diagenetic (i.e., postdepositional) processes, we would expect concentrations to be correlated to organic matter and iron oxides, both present in the pots’ matrix. Coating of clay with organic compounds has been shown to largely increase Hg accumulation [75]. Nevertheless, Kaal et al. [76] found that the organic matter of GUI pottery was dominated by pyrogenic products (fatty acids, polyalkyl-aromatic compounds and polycyclic aromatic hydrocarbons), likely formed upon firing. This suggests that at least part of the Hg that was bound to the soil organic matter, eventually incorporated into the pots in their elaboration, may have been thermally desorbed. Added to this, pyrogenic organic products contain a large abundance of aromatic and carbonyl functional groups, with low efficiency for mercury binding [49,50]. Mercury could have been already present in the clays used as pastes for pottery manufacture. This aspect is discussed in detail below in Section 3.3.

### 3.2. Colour and Mercury Content

All analysed samples fall in a main trend of changes in red and yellow colour components (a* and b*), indicating a shared basic colour in which the main difference between them is in chromatic intensity. Only a few samples, mostly of Plain Penha, showed a redder component (Figure 1). Similar results were reported in a previous study of GUI pottery and Bell Beaker pottery from NW Spain using the CIELab colour space [76,77]. Values for the colour components were in the same range as ours, but L* values of the Penha-type pottery analysed by us tend to be higher (35–66) than those (21–51) described by these authors. Therefore, Penha-type pottery colour was lighter (higher L* values) than Bell Beaker pottery for the same area, even for the Bell Beaker imitation pots. In these studies, lightness (L*) was also negatively correlated to C content, but the correlation was much higher than that observed in our investigation. Kaal et al. [76] also found that the abundance of organic pyrogenic (polycyclic aromatic hydrocarbons) products was negatively correlated to lightness.

Recent studies have linked reddening (a* values) to the maximum temperature reached by fired materials [78,79,80,81] with a linear response until 900 °C [82,83]. These investigations suggest that redness can be used as a temperature indicator of the fired materials, as proposed by Sui et al. [83]. Nevertheless, in the samples studied here, neither redness (a*) nor any of the other colour parameters (L* and b*) showed significant correlations to Hg. This result is consistent with previous studies on the colour of GUI pottery and Bell Beaker pottery of NW Spain [76,77], which showed that pottery colour depended on the original colour of the raw materials, the elemental composition of the clay (i.e., iron content), and interactions between composition and processing (i.e., firing conditions). Thus, redness is not a reliable temperature proxy for pottery elaborated with clays sourced from different types of weathering products (felsic vs. mafic).

### 3.3. Kaolinite Transformations and Mercury Content

Previous investigations have shown that mercury content in soils is correlated with clay content [84], and that Hg concentrations increase by one to two orders of magnitude from coarser fractions to finer soil fractions [85]. Very high Hg concentrations, >1000 ng g^−1^ (up to 14400 ng g^−1^), were found, for example, in topsoils from Europe [46] and mangrove soils enriched in fine fractions [86]. Experimental studies have also shown that kaolinite has a large affinity for metals [87,88,89], including Hg [84,90,91]. Maximum Hg adsorption concentrations exceed 10 mg g^−1^ (1.0 × 10^7^ ng g^−1^), although this depended on pH and the presence of other ions (metals in particular) [92,93]. Experimental studies demonstrated that isomorphic substitution by Mg, pervasive in kaolinite minerals, provides thermal stability and results in strong Hg adsorption [94]. Thus, higher Hg concentrations would be expected for pottery samples from sites located in areas dominated by mafic lithologies, since Mg content in these pots is much higher than in those from felsic areas [72] and Mg substitutions are more likely. The clays used as pastes for pottery manufacture already contained Hg, although no Hg minerals have been identified in the local bedrock where the sites are located. This suggests that temper did not contribute to Hg concentrations in the pots. Therefore, our interpretation is that the actual Hg concentration of the pots was determined by the initial Hg concentration of the clays used as pastes and by firing conditions (temperature and duration), and later use of the pots. At least part of the Hg may have been thermally desorbed during firing of the pots or even further released through repeated heating in vessels used for cooking.

MIR results indicate that the paste of the analysed pottery samples was mainly composed of kaolinite. In a recent investigation that included the sites studied here [72], kaolinite was found in the weathering products of all geological materials, while the primary minerals of the temper differed depending on the dominance of mafic or felsic materials in the surrounding areas. In our samples, a trend in kaolinite transformation was also identified (Figure 4). A few samples (9%) retained well-preserved kaolinite, 20% of the samples showed extensive transformation, and 71% exhibited a complete dehydroxylation of kaolinite. This sequence suggests that surface hydroxyls (absorbance at 3692 cm^−1^) are more easily lost than inner hydroxyls (absorbances at 3621 and 909 cm^−1^). Experimental thermal treatment of kaolinite showed that it starts to collapse at 500–550 °C and progresses depending on increasing temperature and time of treatment: dehydroxylation occurs at lower durations, from 2 h to 10 min, when temperature increases from 500 °C to 600 °C (53–55), and after 3 h at 750 °C it completely transforms into metakaolinite [20,95,96,97,98,99].

In our study, we have found that as kaolinite is progressively dehydroxylated, the intensity of absorbances related to adsorbed water (3400–3200, 1650 cm^−1^) increases, as reflected by the tKAO index. tKAO values differed significantly between archaeological sites and, although the overall correlation with Hg concentration is not significant, the distribution of the samples in the Hg/tKAO projection follows a pattern of decreasing concentration with increasing tKAO that is consistent with Hg thermal desorption (Figure 6A). Estimated initial Hg concentrations varied between 37 and 5515 ng g^−1^, implying Hg losses of 13–99%. The maximum estimated initial Hg concentration is greater than that estimated for the clay fraction of soils from NW Spain (750–1500 ng g^−1^) but within the range of the highest values found for topsoils in other studies [46,86]. The proportion of estimated Hg lost showed large differences between archaeological sites (Figure 6B). Sites such as ZAR and AMM showed very large losses, suggesting higher temperatures, longer firing times and, possibly, more controlled firing conditions. On the other hand, sites such as MON and REQ showed large variations in Hg losses, indicating more variable, and perhaps less controlled, firing conditions.

## 4. Materials and Methods

### 4.1. Material

**Figure 7:**
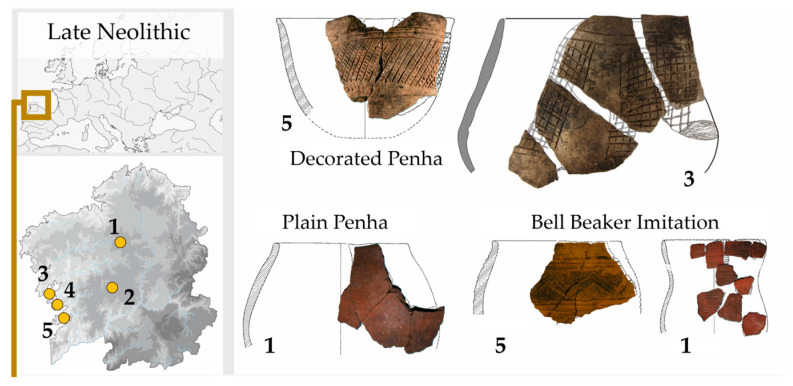
Location of the sites studied and examples of representative subtypologies of Penha pottery. Sites in the map and examples are numbered as follows: 1: REQ, Requeán; 2: ZAR, Zarra de Xoacín; 3: GUI, Guidoiro Areoso; 4: AMM, As Mamelas; 5: MON, Montenegro.

For this investigation, we used 92 Late Neolithic (c. 3100–2400 cal BC), Penha-type pottery samples recovered in five archaeological sites from Galicia (NW Spain) (Table 2, Figure 7). The sites are representative of funerary and settlement contexts and are located in areas of contrasting geological materials: mafic (REQ and ZAR) and felsic (AMM, GUI. MON). Abundant archaeological information is available for the sites as well as the macroscopic characterization of these pots [68,100,101,102,103,104]. A small fragment of each vessel was thoroughly cleaned with ultrapure water, finely milled (<50 µm) and homogenised before analysis.

### 4.2. C, S, and Fe Content

Total carbon and sulfur content of the samples was determined using a LecoTruspec CHNS elemental analyser hosted at the RIAIDT facilities of the Universidade de Santiago de Compostela. Sample replicates were also analysed, and reproducibility was better than 5%, while the detection limit was 0.01% for C and 0.005% for S. Iron concentrations were determined by ICP-MS for a recent investigation that included the pottery samples of this study; full methodological details are provided there [72].

### 4.3. Mercury Analyses

Mercury concentrations were determined by cold-vapour atomic absorption spectrometry (CV-AAS), thermal decomposition and gold-trap amalgamation, using a DMA-80 (Milestone^™^ SpA, Sorisole, Italy). The equipment is located at the EcoPast research group facilities at CRETUS Institute, Universidade de Santiago de Compostela. Analytical quality control was ensured through the routine use of procedural blanks and standard reference materials (SRMs). Each analytical sequence comprised three initial blanks and two SRMs, followed by the periodic analysis of one blank every four samples and two SRMs every fifteen samples to monitor and ensure instrumental stability and analytical performance.

The method limit of quantification (LOQ), established at 0.69 ng g^−1^ from procedural blanks, defined the lower limit of reliable quantification. Analytical accuracy was evaluated using the following standard reference materials: BCR 277R (128 ± 17 ng g^−1^; estuarine sediment) and NIST 1570a (29.70 ± 2.10 ng g^−1^; spinach leaves), yielding mean recoveries of 104% and 71%, respectively. Duplicate analyses were performed for 45% of the samples, resulting in a coefficient of variation of 2.9%. Mercury analysis is a micro-destructive technique (~20 mg of sample) that enables minimizing the impact on archaeological objects, something we must always consider when dealing with archaeological heritage.

### 4.4. Colour (CIELab Space)

Colour was determined using a Konica-Minolta CR-R Chroma Meter for solids (Konica Minolta Inc., Tokyo, Japan) on finely ground and homogenised samples. Colour was expressed in the CIELab three-dimensional space, defined by lightness (L*) and colour components (a* and b*). Component a* varies between green (negative values) and red (positive values), while component b* indicates blue (negative values) to yellow (positive values). CIELab colour system is widely used in both the scientific community and industry, as it represents the most uniform colour space from a perceptual point of view [105,106].

### 4.5. Clay Transformations (Kaolinite and Talc)

Finely milled samples were also analysed using Fourier-transform infrared spectroscopy with attenuated total reflectance (FTIR-ATR) in the mid-infrared (MIR, 4000–400 cm^−1^). We used a Cary 630 spectrometer (Agilent Technologies Inc., Santa Clara, CA, USA), located at the EcoPast research group facilities (Universidade de Santiago de Compostela, Spain). Spectra were collected using a resolution of 4 cm^−1^ and performing 100 scans per sample to improve reproducibility and signal-to-noise ratio. Spectral processing was done with Orange data mining software v3.40.0 [107].

Based on the results obtained, we calculated a MIR index accounting for the structural transformations of kaolinite. This index is based on the decrease in intensity of the main Si-O vibrations of kaolinite (at 1002 cm^−1^) and the increase in intensity of the absorbance associated with adsorbed water (3400–3200 cm^−1^):


tKAO: maximum abs at 1002 cm^−1^/maximum abs at 3400–3200 cm^−1^ (1)

We also calculated the ratio based on area below 3400–3200 cm^−1^ and 1200–850 cm^−1^, but the results of both indices were highly correlated (r^2^ = 0.98, *p* < 0.01) and thus provide the same information for the set of samples studied here.

### 4.6. Statistical Analyses

Concentration data for C, S, and Fe, in µg g^−1^, and Hg, in ng g^−1^, were log-transformed before performing statistical tests. ANOVA was applied to check for differences between archaeological sites and Penha-type pottery styles. Pearson correlation coefficient was used to assess covariation.

## 5. Conclusions

The primary finding of this study is the presence of mercury in detectable concentrations in all analysed pottery samples; an unexpected result given the volatile nature of the element. Mercury concentrations varied considerably across sites and between individual vessels of the same site, suggesting that these differences may be archaeologically significant and/or related to pre- and post-depositional processes. This raises the question of when and by what mechanisms mercury became incorporated into the ceramics. The incorporation could have occurred at any point in the vessel’s life history: selection of raw material, production, use, re-use or disposal [2].

Since mercury has a high affinity to bind to organic matter, iron oxides, or be adsorbed onto clay minerals, each of these potential pathways was evaluated separately. Regarding organic matter, no correlation was found. Moreover, based on previous studies, the preserved organic fraction seems to be mostly composed of pyrogenic products, suggesting that Hg incorporation occurred during the ceramic production rather than through post-depositional processes, and later burning upon firing. This result is interpreted as a lack of evidence of mercury incorporation through diagenetic processes mediated by organic matter (lack of correlation with C and S). Likewise, if mercury was present in the organic component of raw materials, it may have been released during firing. A similar result is observed for iron oxides, as no correlation has been found with either total iron or colour, with redness being an indicator of both iron oxides and firing temperature.

A stronger relationship seems to exist with clay; specifically, higher concentrations tend to occur in pots in which kaolinite showed lower thermal-induced transformation. In fact, mercury concentration and its variability decreased with increasing kaolinite transformation. These thermal transformations of kaolinite are connected to the potter’s choices when making a vessel, selection of raw materials, tools, energy, techniques and their sequence [17]. Therefore, mercury may have been already present in the clay or could have been incorporated into it (i.e., adding cinnabar) and later desorbed during firing. Some chemical processes, such as Mg isomorphic substitutions, could have influenced kaolinite stabilization and resistance to thermal transformation, enhancing mercury retention—in ceramics from mafic areas in particular. Furthermore, this is consistent with the use of local raw materials in the production of the ceramics observed in a previous study [72]. Note also that firing conditions seem to affect all those processes. The firing technology using simple kilns, as those typical of Late Neolithic communities, leads to poor control of temperature and time, resulting in large differences among vessels that could explain the significant variability observed in the Hg content. Further investigation would seek to address pottery from other periods and areas, specifically those from cultures less obsessed with cinnabar use. Moreover, experiments incorporating different ceramic compositions and firing conditions, inspired by the work of Gliozzo [20], would provide a more robust assessment of mercury content.

In conclusion, this first research into the mercury content in pottery leaves us with several open questions. One obvious issue is the potential influence of the widespread use of mercury and cinnabar by Late Neolithic societies, well documented in funerary contexts elsewhere [59,64]. The fact that ceramics with Bell Beaker imitation decoration showed higher mercury concentrations than Penha decorated, and both higher than Plain Penha ones, could be associated with the described social interaction of cinnabar with the Bell Beaker culture [65]. However, there is no relationship between Hg and S (expected if cinnabar was present) to support this hypothesis. Also, pottery from funerary deposits does not show higher Hg concentrations than those from settlements. Simply, further research is required to clarify the mechanisms underlying mercury incorporation. At least for now, the most parsimonious explanation is that mercury content was controlled by the initial concentrations in the clays and later desorption during firing. Whether the use of mercury-enriched clays originally stemmed from unintentional decisions or was a decision linked to the Chaîne Opératoire remains to be explored. In any case, analysing mercury in prehistoric pottery opens up a new field of research with great potential for the future of Archaeology.

## Figures and Tables

**Table 1:** Correlation between the chemical elements and the colour parameters of the Late Neolithic Penha-type pottery samples of this study. L*, lightness; a*, redness; b*, yellowness.

	S	Fe	L*	a*	b*	Hg
C	0.29	−0.07	−0.58	−0.46	−0.52	0.09
S		−0.13	0.13	−0.08	0.05	−0.07
Fe			−0.01	0.47	0.43	−0.10
L*				0.51	0.75	−0.13
a*					0.92	−0.29
b*						−0.29

**Table 2:** Archaeological sites, periods, type of archaeological context, and number of pottery samples analysed. Although most sites expand beyond the Neolithic, the analysed pottery samples were all Penha-type and are assigned to the Late Neolithic. LN, Late Neolithic (c. 3100–2400 cal BC); LBA, Late Bronze Age (c. 1200–800 cal BC).

Site Name	Chronology	Code	N. Samples	Type of Context	Reference
Requeán	LN	REQ	21	Settlement	[101]
Zarra de Xoacín	LN–LBA	ZAR	17	Settlement	[102]
As Mamelas	LN–LBA	AMM	10	Settlement and funerary	[103]
Guidoiro Areoso	LN–LBA	GUI	9	Funerary	[104]
Montenegro	LN–LBA	MON	35	Settlement	

## Data Availability

Most data used to produce this research are included in the main text and Appendix A, as well as in Supplementary Material here and in Supplementary Material in Martínez Cortizas et al. [72].

## Supplementary Materials

The following supporting information can be downloaded at: https://www.mdpi.com/article/10.3390/molecules31132335/s1, A file containig carbon, sulfur, and Fe content, as well as the color parameters (L*, a*, and b*).

## Appendix A

**Table A1:** Average, standard deviation, maximum, and minimum values for the elemental composition and colour parameters of the studied pottery samples. Carbon, S and Fe in percentage; Hg in ng g^−1^. L*, lightness; a*, redness; b*, yellowness.

Site		C	S	Fe	Hg	L*	a*	b*
REQ	avg	2.31	0.07	5.43	60	45.1	8.3	17.4
	std	0.82	0.04	1.36	55	5.1	5.0	6.2
	max	4.35	0.18	8.57	176	53.4	20.1	27.3
	min	1.22	0.01	2.36	12	37.4	3.1	7.7
ZAR	avg	1.85	0.08	4.89	36	45.3	9.1	18.1
	std	0.61	0.03	1.90	33	5.4	4.7	6.5
	max	2.77	0.15	7.15	127	52.5	17.4	27.2
	min	1.09	0.02	0.52	9	36.4	2.9	7.9
AMM	avg	2.61	0.20	2.89	30	45.7	5.8	15.2
	std	0.61	0.08	1.90	12	8.5	3.1	5.9
	max	4.00	0.30	4.68	47	66.1	10.7	25.4
	min	2.01	0.03	0.83	10	37.4	0.7	8.1
GUI	avg	1.68	0.05	2.46	174	43.0	4.4	11.3
	std	0.51	0.03	1.28	344	7.5	2.0	2.8
	max	2.27	0.11	5.07	1086	57.7	8.9	17.3
	min	0.95	0.01	0.87	13	35.2	2.4	9.1
MON	avg	2.04	0.14	2.59	99	48.6	6.4	16.2
	std	0.82	0.08	0.82	110	7.7	3.3	5.9
	max	4.49	0.31	5.23	381	62.8	14.8	26.8
	min	1.18	0.01	0.91	6	37.9	2.6	7.2
Decorated	avg	2.06	0.11	3.44	98	45.9	6.3	15.2
	std	0.73	0.08	1.65	174	6.2	3.1	5.4
	max	3.68	0.30	7.02	1086	58.1	14.8	26.8
	min	0.95	0.01	0.83	9	35.2	0.7	7.2
Plain Penha	avg	1.99	0.12	3.87	40	47.8	8.8	18.6
	std	0.73	0.08	1.97	45	8.0	4.8	6.6
	max	4.00	0.31	8.57	176	66.1	20.1	27.3
	min	1.09	0.01	0.52	6	36.7	2.8	7.7
Imitation	avg	2.47	0.10	4.04	107	44.3	5.6	13.9
	std	0.94	0.06	1.86	94	6.6	4.2	5.3
	max	4.49	0.22	7.57	351	57.0	19.2	27.2
	min	1.29	0.03	1.78	12	36.5	2.4	7.7
